# Evaluation of the Cellsway Microfluidic CTC Enrichment and Identification Platform for CTC Detection in Metastatic NSCLC

**DOI:** 10.3390/bios16010034

**Published:** 2026-01-02

**Authors:** Ebru Özgür, Ayça Çırçır, Begüm Şen Doğan, Şebnem Şahin, Gizem Karayalçın, Mehmet Alper Demir, Başak Erkek, Enes Demirtaş, Özge Zorlu, Furkan Ceylan, Haluk Külah, Nuri Karadurmuş, Mehmet Ali Nahit Şendur, Saadettin Kılıçkap

**Affiliations:** 1 Mikro Biyosistemler A.S., 06530 Ankara, Türkiye; ayca.circir@mikrobiyo.com.tr (A.Ç.); gizem.karayalcin@mikrobiyo.com.tr (G.K.); ozge.zorlu@mikrobiyo.com.tr (Ö.Z.);; 2 Department of Micro and Nanotechnology, Middle East Technical University, 06800 Ankara, Türkiye; 3 Department of Medical Oncology, Ankara Bilkent City Hospital, 06800 Ankara, Türkiye; 4 METU MEMS Center, 06530 Ankara, Türkiye; 5 Department of Electrical and Electronics Engineering, Middle East Technical University, 06800 Ankara, Türkiye; 6 Department of Medical Oncology, University of Health Sciences, Gülhane Training and Research Hospital, 06010 Ankara, Türkiye; 7 Department of Medical Oncology, Yıldırım Beyazıt University, 06800 Ankara, Türkiye; 8 Department of Medical Oncology, Faculty of Medicine, İstinye University, 34408 İstanbul, Türkiye; 9 Medical Oncology Unit, Liv Hospital Ankara, 06680 Ankara, Türkiye

**Keywords:** circulating tumor cells (CTC), non-small-cell lung cancer (NSCLC), liquid biopsy, microfluidics, size-based separation

## Abstract

Lung cancer is the leading cause of cancer-related mortality worldwide, with non-small-cell lung cancer (NSCLC) accounting for the majority of cases. Standard tissue biopsies are invasive and unsuitable for repeated monitoring. Liquid biopsy technologies, particularly circulating tumor cell (CTC) analysis, offer a minimally invasive alternative for real-time disease tracking. To address the need for efficient and reproducible CTC isolation, we developed the Cellsway microfluidic CTC enrichment and identification platform, which employs inertial hydrodynamics in a spiral-shaped microfluidic channel comprising hydrofoil-shaped pillars to enable high-throughput, label-free enrichment of CTCs while preserving cell integrity, followed by an optimized CTC identification assay. Analytical performance assessed through spiking experiments using NSCLC cell lines demonstrated recovery rates of 91.9% for H1975 cells and 78.3% for A549 cells. Clinical validation was performed on blood samples from 51 stage IV NSCLC patients. A 7.5 mL volume of peripheral blood was processed with the SwayBox platform, and enriched CTCs were identified through an optimized multiplex immunofluorescence protocol. CTCs were detected in 47% of NSCLC patients, with counts ranging from 0 to 72 cells per 7.5 mL of blood. At a cutoff of 1 CTC per 7.5 mL, the assay achieved a specificity of 95%. Patient-derived CTCs exhibited smaller mean diameters compared to cultured NSCLC cell lines, yet were effectively enriched through hydro-dynamic tuning. These findings demonstrate that the Cellsway platform enables efficient and re-producible CTC isolation with high specificity, supporting its potential utility for clinical monitoring and precision oncology in NSCLC.

## 1. Introduction

Lung cancer is the leading cause of cancer-related deaths globally, accounting for the highest mortality rates among both women and men [1]. According to GLOBOCAN, in 2022, there were over 2.48 million new cases and 1.8 million deaths worldwide. Lung cancer is typically diagnosed at advanced stages, as early symptoms are often mild and easily mistaken for common respiratory condition, thereby complicating disease management and contributing to high mortality rates [2,3]. Lung cancer has been classified into two histological subtypes as small-cell lung cancer (SCLC) and non-small-cell lung cancer (NSCLC). NSCLC accounts for 85% of all lung cancer cases and comprises mainly 3 subtypes as adenocarcinoma (50–60%), squamous cell carcinoma (20–30%) and large cell carcinoma (10–20%) [4]. Smoking is the major risk factor for lung cancer but there is also an increase in lung cancer incidences among non-smokers due to air pollution and exposure to environmental carcinogens [5].

Over the past two decades, significant advances in the treatment of NSCLC have markedly improved the overall survival (OS) and progression-free survival (PFS) for both early- and advanced-stage patients. In the current cancer treatment landscape—characterized by a growing array of targeted therapies, immune checkpoint inhibitors (ICIs), and advanced treatment modalities, continues monitoring of disease evolution is essential to enable timely and effective therapeutic intervention [6]. This underscores the need for careful patient stratification and a precision diagnostics approach to ensure optimal treatment selection and effectiveness. Standard tissue biopsy is highly invasive and poses risks of complications, making it unsuitable for frequent use during treatment and disease monitoring. Additionally, it offers only a static and spatially limited snapshot of the tumor’s genetic landscape. In many cases, tumor tissue may be inaccessible or insufficient in quantity to perform comprehensive molecular analysis [7,8].

Liquid biopsy technologies have emerged as a powerful alternative tool for non-invasive and real-time tracking of disease progression and therapeutic response. They enable the detection of tumor-derived components, including circulating tumor cells (CTCs), tumor-free DNA (cfDNA), extracellular vesicles (EVs), and tumor-educated platelets (TEPs), from a simple blood sample, facilitating dynamic assessment of tumor evolution, early identification of resistance mechanisms, and timely adaptation of treatment strategies [9,10,11,12]. Among these technologies, CTC- and ctDNA-based analyses have become clinically available, particularly for monitoring of treatment response and tracking genomic alterations [13]. In the management of NSCLC, ctDNA analysis is mostly employed for monitoring tumor burden, detecting minimal residual disease (MRD), identifying targetable mutations and genomic alterations [14,15,16,17]. However, current assays have limited sensitivity in early-stage disease, in identification of low-frequency mutations, in detection of MRD, and in predicting response to ICI treatment [18,19,20,21,22]. These limitations are further complicated by clonal hematopoiesis of indeterminate potential (CHIP), which can affect interpretation and lead to false-positive results [21,23]. The main limitation is that ctDNA analysis does not provide information on clinically relevant expression level (RNA or protein) variations—e.g., PD-L1, HER2.

On the other hand, CTC analysis provides multi-omics analyses, including genomic, transcriptomic, and proteomic profiling, that enhances understanding of tumor biology and evolution [24,25]. Prognostic and predictive value of CTCs in monitoring disease prognosis and predicting recurrence in early- or advanced-stage NSCLC patients has been well documented by clinical studies using different CTC isolation platforms [24,25,26,27,28,29,30]. Meta analysis of 18 studies (1321 patients) demonstrated that baseline and prospective CTC positivity is strongly associated with poorer survival and disease-free survival in resectable NSCLC [26]. Li et al. has explored the value of preoperative CTC concentration in predicting postoperative metastasis and recurrence risk in patients with stage I-IIIA NSCLC patients [28]. The results showed that a higher preoperative CTC level independently predicted the risk of metastasis. Similarly, persistent CTC detection before and after radiotherapy has been shown to be correlated with increased risk or regional and distant recurrence [31]. Beyond CTC enumeration, additional insights that can be gained from CTC-based liquid biopsy includes the ability to track epithelial-mesenchymal transition (EMT) status, monitor PD-L1 status for predicting immune therapy efficacy, asses phenotypic and genotypic heterogeneity at the single cell level, and perform functional drug testing assays in preclinical settings—provided that CTCs are isolated in a viable form [32,33,34,35,36].

CTC isolation technologies developed to date have primarily focused on two strategies, (i) immunoaffinity-based separation relying on tumor-specific surface markers, (ii) label-free separation leveraging biophysical differences between CTCs and blood cells, which include size, deformability and electrical properties [29,37,38]. Due to their extreme rarity (1–100 CTCs/mL blood, on average), versatility, fragility, dynamic nature and heterogeneity, it is highly challenging to isolate CTC in high sensitivity and specificity from blood. To overcome this barrier, microfluidic platforms, designed to utilize forces acting at the scales of a cell, have gained a lot of interest in CTC isolation. Utilizing different principles of separation, various microfluidic platforms have been designed, offering notable advantages in sensitivity, specificity, recovery efficiency, and the preservation of cell integrity and viability [39,40,41]. Among these, label-free inertial microfluidic approaches that harness microscale hydrodynamic forces such as pinched flow fractionation [42], deterministic lateral displacement [43], or curved microchannels, offer additional benefits, including higher throughput and ability to capture heterogeneous CTC populations, irrespective of the surface marker expression. Particularly, spiral-shaped microfluidic channels have been widely employed for size-based separation of CTCs from blood cells. These microfluidic designs exploit the balance between the Dean and lift forces exerted on the particles, positioning them at different locations along the width of the microchannel. Efforts on improving the separation efficiency of these designs include utilizing a sheath flow [44] or a secondary flow direction [45]. Geometric enhancement in channel topology and cross-sectional architecture rather than completely relying on curved channel-induced Dean flows have also been studied by utilizing a trapezoidal cross-section [46,47] and sharp turns [48,49] on the microfluidic path. All these designs focus on increasing the separation efficiency in the expense of either one or more of more complicated operation, more difficult manufacturing, larger chip footprint, or increased microfluidic path, and increased forces exerted on the cells, which may disturb cell intactness and viability. We have reported a microfluidic design incorporating a hydrofoil structure at the downstream of a 4-turn spiral microfluidic channel having a regular rectangular cross-section with no sheath flow requirement, showing high CTCs enrichment efficiency while maintaining cell intactness and viability [50,51]. Preliminary clinical performance evaluation of the microfluidic technology in early-stage prostate cancer patients demonstrated significantly higher CTC positivity rates compared to reported studies, while no CTCs were detected in healthy control cohort [52]. Later, the microfluidic design has been improved in terms of shortened microfluidic path and increased separation efficiency by gently widening the outlet region of the microfluidic channel and utilizing successive hydrofoil structures. This design has been analytically validated with different cell lines spiked into blood samples from healthy donors [53].

Cellsway microfluidic CTC enrichment and identification platform investigated in this study performs label-free, size-based isolation of CTCs from blood in a high-throughput manner. The technology employs the microfluidic chip (SwayChip) reported in [53] (Figure S1). The SwayChip is operated by a fully automated, compact, benchtop instrument (SwayBox, Figure S2). The isolated cells are then stained by an in-house optimized CTC identification assay (SwayLights) for analysis.

In this study, we evaluated the analytical and clinical performance of the Cellsway platform for the enrichment of CTCs from NSCLC patients. Spiking experiments have been performed using NSCLC cell lines for determination of analytical performance characteristics, including CTC recovery rates. Clinical performance has been evaluated using blood samples from 51 metastatic NSCLC patient blood samples, and 20 healthy controls. Enriched CTCs have been identified through a downstream immune fluorescence (IF) assay (SwayLights), optimized for the Cellsway platform.

## 2. Materials and Methods

### 2.1. Patients Eligibility

Clinical study has been designed as multi-center, prospective, observational study designed to assess the clinical performance of the Cellsway platform in patients with stage IV non–small-cell lung cancer (NSCLC) and healthy donors. The study has been ongoing since January 2024 and enrolled both male and female participants aged 18 years and older. Patients with histopathologically confirmed stage IV NSCLC at their initial diagnosis or relapsed patients with radiologically confirmed tumors were included. Patients under active chemo/radio/immunotherapy has not been included in performance evaluation study. Written informed consent was obtained from all participants prior to blood collection. Healthy individuals with no prior history of malignancy served as negative controls. All study procedures were granted by the Ethical Committee of Ankara Bilkent Şehir Hastanesi, Ankara, Turkey, (Protocol No: MBS-CTC-HEU-03, Date: 26 May 2023). All procedures were performed in accordance with institutional ethical guidelines and the principles of the Declaration of Helsinki.

A total of 57 NSCLC patients were recruited. Of these, 3 did not meet the inclusion criteria and 3 were excluded due to unreliable experimental results. In total, 51 patients were included in the analysis. Among them, 35 were diagnosed with adenocarcinoma (ADC), 12 with squamous cell carcinoma (SCC), and 4 with NSCLC not otherwise specified (NOS). Twenty age-matched healthy volunteers were included as controls to evaluate the sensitivity and specificity of the platform. Exclusion criteria comprised recent surgical intervention or systemic chemotherapy before CTC analysis, comorbidities incompatible with study participation, absence of biopsy-confirmed stage IV NSCLC, delayed blood processing beyond 4 h, insufficient blood volume (<6 mL), or macroscopic abnormalities such as clotting, hemolysis, or air contamination. Individuals or samples meeting any of these criteria were excluded from the final analysis.

For spiking experiments to assess analytical performance, healthy volunteers’ blood samples were collected at Middle East Technical University Medical Center following the protocols approved by the Ethical Committee of Ankara Şehir Hastanesi, Ankara, Turkey (Protocol no: MBS-CTC-03, Date: 27 December 2023). Written informed consent was obtained from all participants prior to blood collection.

### 2.2. Sample Collection

Peripheral blood samples were collected in 10 mL K2-EDTA vacutainer tubes (Becton Dickinson, Mississauga, ON, Canada). To minimize the risk of epithelial cell contamination during venipuncture, the first 2 mL of blood was collected into a separate tube and discarded. Subsequently, 7.5 mL of blood was obtained for downstream analyses. All samples were transported to the laboratory in a dedicated blood transport container and processed within 4 h of collection to ensure optimal cell viability. During interim storage, samples were maintained at room temperature until processing.

### 2.3. Cell Culture

Cultured human NSCLC A-549 and H-1975 cell lines were kindly provided by Prof. Petek Korkusuz (Hacettepe University) and METU MEMS Center, respectively. All cell lines were cultured at 37 °C in 5% CO_2_. A-549 cell line was cultured in a growth medium containing Dulbecco’s Modified Eagle’s Medium—High Glucose (DMEM-HG) (VivaCell Biosciences, Denzlingen, Germany), 10% fetal bovine serum (FBS) (Serana Europe GmbH, Pessin, Germany), 2 mM L-Glutamine Solution 100X (Serana, Pessin, Germany) and 1% penicillin–streptomycin (VivaCell Biosciences). H-1975 cell line was cultured with RPMI-1640 medium (Biological Industries, Kibbutz Beit HaEmek, Israel), 10% FBS, 1% penicillin–streptomycin. Cells were subcultured at 70–80% confluency, and then they were dissociated using 0.25% trypsin-EDTA (VivaCell Biosciences) and resuspended in phosphate-buffered saline (PBS) solution.

### 2.4. Spiking Experiments

For analytical performance evaluation, spiking experiments were carried out with cancer cells fluorescently labeled with Cell Tracker Red CMTPX Dye (Invitrogen, Waltham, MA, USA). The cell concentration was measured with a TC20 automated cell counter (BioRAD, Hercules, CA, USA). Trypan blue was used to measure cell viability. To achieve the desired cell number in spiking experiments for analytical performance evaluation studies, cells were serially diluted to obtain the desired concentration (100 cells/1 mL PBS) and spiked into whole blood (7.5 mL) collected from healthy donors.

### 2.5. Blood Pre-Processing

Peripheral blood mononuclear cells (PBMCs) were isolated using a modified density gradient centrifugation protocol with Secoll (Serana Europe GmbH). Briefly, whole blood was diluted with cell suspension solution and carefully layered over Secoll medium in a 50 mL conical tube, taking care to avoid mixing the two phases. Centrifugation was performed in a swing-out rotor with brake-off mode to maintain gradient integrity. After separation, the plasma layer was partially removed, leaving sufficient volume to prevent disturbance of the PBMC interface. The PBMC fraction was then aspirated together with a minimal portion of underlying density medium to ensure complete recovery and transferred into a sterile conical tube. Cells were washed twice with excess volumes of FBS-PBS solution to remove residual plasma proteins and density medium. The final pellet was resuspended in fresh FBS-PBS solution and immediately processed by the SwayBox microfluidic platform.

### 2.6. Cellsway Microfluidic CTC Enrichment Platform

Cellsway CTC enrichment platform is a microfluidic system comprising a microfluidic chip (SwayChip) (Mikro Biyosistemler A.Ş., Ankara, Türkiye) for size-based CTC isolation and an operating unit (SwayBox) (Mikro Biyosistemler A.Ş., Ankara, Türkiye) designed for automated processing of blood samples through SwayChip (Figure S2). SwayChip is comprised of a spiral microfluidic channel with one inlet and two outlets (the Product and Waste outputs) and utilizes hydrodynamic enrichment principles to isolate circulating tumor cells (CTCs) based on their size and deformability. The microfluidic channel is composed of a 2-loop Archimedean spiral part which is followed by a gently enlarging outlet region which includes hydrofoil-shaped pillars before the microfluidic channel splits into two branches (Product and Waste). The Archimedean spiral focuses the particles to their respective positions according to their sizes within a minimum travel distance in the microfluidic channel. Successively placed asymmetric hydrofoils in the widening section of the channel enhances the separation distance between different-sized particles by forcing the larger particles towards the inner wall (guiding them to the Product outlet) of the microfluidic channel, and smaller ones to the outer wall (to the Waste outlet). Widening of the outlet region provides more room for positioning of the hydrofoils and decreases the forces acting on the cells. Overall, the microfluidic channel structure enhances the separation efficiency, shortens the length required for hydrodynamic size-based enrichment, thereby minimizing the damage resulting from shear stress. The patented microfluidic channel design principles and analytical validation data have been previously reported [50,51,53].

Prior to sample processing, the SwayChip was preconditioned through sequential washing by ethanol (EtOH), deionized (DI) water and phosphate-buffered saline (PBS). After that, samples were introduced into the chip at a constant flow rate of 1.2 mL/min, which was continuously monitored through and verified by a flow sensor embedded in the platform. The product output, enriched for circulating tumor cells (CTCs), and waste output were collected for downstream calculations and analyses.

WBC depletion was assessed for both patient and healthy donor samples by comparing the white blood cell concentration in the inlet and product samples. Depletion rates were calculated using the following equation:

(1) $$\mathrm{WBC}\;\mathrm{depletion}\;\mathrm{rate}\;(\%)=1-\frac{\#\mathrm{of}\;\mathrm{WBCs}\;\mathrm{at}\;\mathrm{product}\;\mathrm{outlet}}{\#\mathrm{of}\;\mathrm{WBCs}\;\mathrm{at}\;\mathrm{inlet}}\times 100$$

In analytical performance evaluation through spiking experiments, CTC recovery rate has been calculated through the following equation:

(2) $$\mathrm{CTC}\;\mathrm{recovery}\;\mathrm{rate}\left(\%\right)=\frac{\#\mathrm{of}\;\mathrm{CTCs}\;\mathrm{at}\;\mathrm{product}\;\mathrm{outlet}}{\#\mathrm{of}\;\mathrm{CTCs}\;\mathrm{at}\;\mathrm{product}\;\mathrm{and}\;\mathrm{waste}\;\mathrm{outlets}}\times 100$$

### 2.7. CTC Identification by Immunofluorescence Analysis

Enriched CTC suspension collected at the product outlet were analyzed for CTC identification with the SwayLights assay (Mikro Biyosistemler A.Ş., Ankara, Türkiye), through multiplex immunofluorescent (IF) analysis. Following enrichment, cells were fixed with 2% formaldehyde (Sigma Aldrich, Darmstadt, Germany), transferred onto poly-L-lysine (PLL)-coated microscope slides, and air-dried. Cells were permeabilized using 0.1% Triton X-100 (Thermo Fisher Scientific, Waltham, MA, USA) for 10 min, and blocking was carried out with 2% bovine serum albumin (BSA) (Thermo Fisher Scientific) in PBST (0.1% Tween-20 in PBS; Sigma Aldrich) for 1 h at room temperature in a humidified chamber.

Cells were incubated overnight at 4 °C with a primary antibody cocktail prepared in blocking buffer. The cocktail contained epithelial markers, including pan-cytokeratin, cytokeratin 8/18 and EpCAM/TROP1 (Novus Biologicals, Centennial, CO, USA), all conjugated with Alexa Fluor 750. A mesenchymal marker, vimentin (Novus Biologicals), also conjugated to Alexa Fluor 750, was included. To exclude leukocytes, two different clones of anti-CD45 antibodies (Novus Biologicals and Abcam, Cambridge, UK) conjugated with Alexa Fluor 647, were used. Following antibody incubation, cell nuclei were counterstained with DAPI (Invitrogen). Slides were subsequently washed with PBST and mounted with Fluoromount aqueous mounting medium (Sigma Aldrich).

Fluorescence imaging was carried out using an automated fluorescence scanning microscope (Bioview AllegroPlus, Rehovot, Israel). CTCs were identified as positive for cytokeratin and/or EpCAM and/or Vimentin (Cy7 channel), negative for CD45 (Cy5 channel), and positive for DAPI.

**Figure 1:**
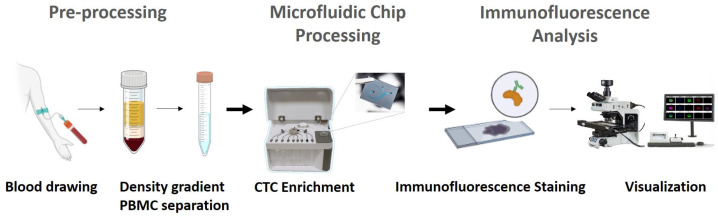
Schematic representation of the CTC enrichment workflow. Peripheral blood samples collected from patients and healthy individuals were processed using a three-phase workflow comprising PBMC isolation, microfluidic enrichment, and immunofluorescence-based analysis. Pre-processing: Following blood collection, peripheral blood mononuclear cells (PBMCs) were isolated via density gradient centrifugation. Microfluidic chip processing: The resulting PBMC fraction was resuspended and introduced into the SwayChip. Leveraging hydrodynamic separation principles, the SwayChip enabled size and deformability-based isolation of circulating tumor cells (CTCs) from other blood components under controlled flow conditions. Immunofluorescence analysis: The enriched output was collected and transferred onto PLL-coated microscope slides for downstream immunofluorescence staining. Fluorophore-conjugated antibodies were applied to identify tumor-associated markers and exclude leukocyte-specific signals (SwayLights). Slides were imaged using an automated fluorescence scanning platform, and CTCs were enumerated based on their immunophenotypic profiles.

The whole workflow for blood processing, CTC isolation and IF-based CTC identification is presented in Figure 1.

### 2.8. CTC and Cell Line Size Measurements

The sizes of identified CTCs were measured via BioView platform, using the measurement scale integrated into the BioView software (version 3.8.1.4), based on their brightfield images. Similarly, the diameters of A549, H1975 and WBCs were assessed after spiking, enrichment and subsequent IF staining, using the BioView system. In addition, WBC dimensions were independently evaluated using size-standardized fluorescent beads as external calibrators. WBCs were mixed with 10 µm and 20 µm diameter beads at optimized ratios and imaged under a Leica DM2000 LED microscope (Danaher Corporation, Washington, DC, USA) at 20X magnification using the LAS EZ software (version 3.4.0). The acquired images were subsequently analyzed in ImageJ (version 1.54g), where bead diameters were repeatedly measured to establish a reference scale and ensure accurate size quantification.

### 2.9. Statistical Analysis

Statistical analyses were performed using Jamovi Statistics software (version 2.6.44) and R Studio (version 2024.12.0). The Shapiro–Wilk test was used to assess the normality of data distributions. Due to non-parametric characteristics of the data, Mann-Whitney test was used to evaluate the relationship between CTC positivity and continuous clinical variables, including age, sex, tumor size, lymph node metastasis and the number of metastatic organs. The association between NSCLC histological subtypes and CTC positivity was analyzed using the Chi-Square test. Associations between categorical variables were evaluated using Fisher’s exact test. Statistical significance was set at *p* < 0.05, with a 95% confidence interval (CI) applied for all analyses.

## 3. Results

### 3.1. Analytical Validation of SwayBox Microfluidics (SwayChip Operated by SwayBox)

To validate the performance of SwayChip and SwayBox, recovery efficiency was assessed using controlled spiking experiments. Previous validation studies conducted at a flow rate of 1500 μL/min with various tumor cell lines had demonstrated the reliability and reproducibility of the system [53]. In the present study, a flow rate of 1200 μL/min was applied in order to improve the recovery rate, and two NSCLC cell lines—H1975 and A549—were selected to better reflect the biological context of clinical specimens.

For each experiment, 100 fluorescently labeled cells were spiked into 7.5 mL of healthy donor blood. After density gradient separation, the resulting PBMC suspension was processed through the SwayChip following the same workflow as applied to patient samples. At 1200 μL/min, recovery rates were achieved as 91.9 ± 7.9% for H1975 and 78.3 ± 18.8% for A549. The difference in recovery rates is consistent with the mean cell diameters of the respective lines, measured as 17.4 ± 4.3 µm for H1975 and 13.4 ± 2.0 µm for A549, indicating that the larger average size of H1975 cells likely contributes to their higher recovery efficiency.

These findings confirm the capability of the SwayChip operated by SwayBox to reproducibly isolate rare tumor cells from whole blood under clinically relevant conditions.

### 3.2. Clinical Performance of Cellsway Platform

**Figure 2:**
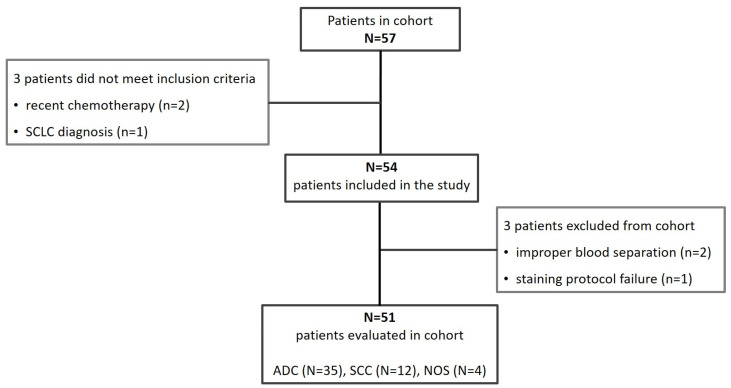
Patient inclusion flowchart.

The study cohort included patients with non-small-cell lung cancer (NSCLC) and healthy donors. All healthy donor samples (N = 20) were retained for analysis. Among NSCLC patients, two samples were excluded at baseline due to different histopathological tumor types and prior chemotherapy, and three were excluded at different phases of the workflow, two because of inadequate blood separation and one for failed staining (Figure 2).

Baseline demographic and clinical characteristics of eligible patients were presented in Table 1 and Table S1. In total, 51 patients with stage IV NSCLC were analyzed. Among them, 36 were male (71%) and 15 were female (29%), with a median age of 64 years (range, 45–82 years). Histologically, adenocarcinoma (ADC) was the most frequent subtype (69%), followed by squamous cell carcinoma (SCC, 24%) and not otherwise specified (NOS, 8%). Tumor volumes ranged from 13 to 120 mm, with a median of 32.8 mm. Bone was the most common metastatic site, and two-organ dissemination represented the most frequent metastatic pattern (57%). Nearly all patients exhibited lymph node involvement, with the number of metastatic lymph nodes varying considerably across the cohort.

**Figure 3:**
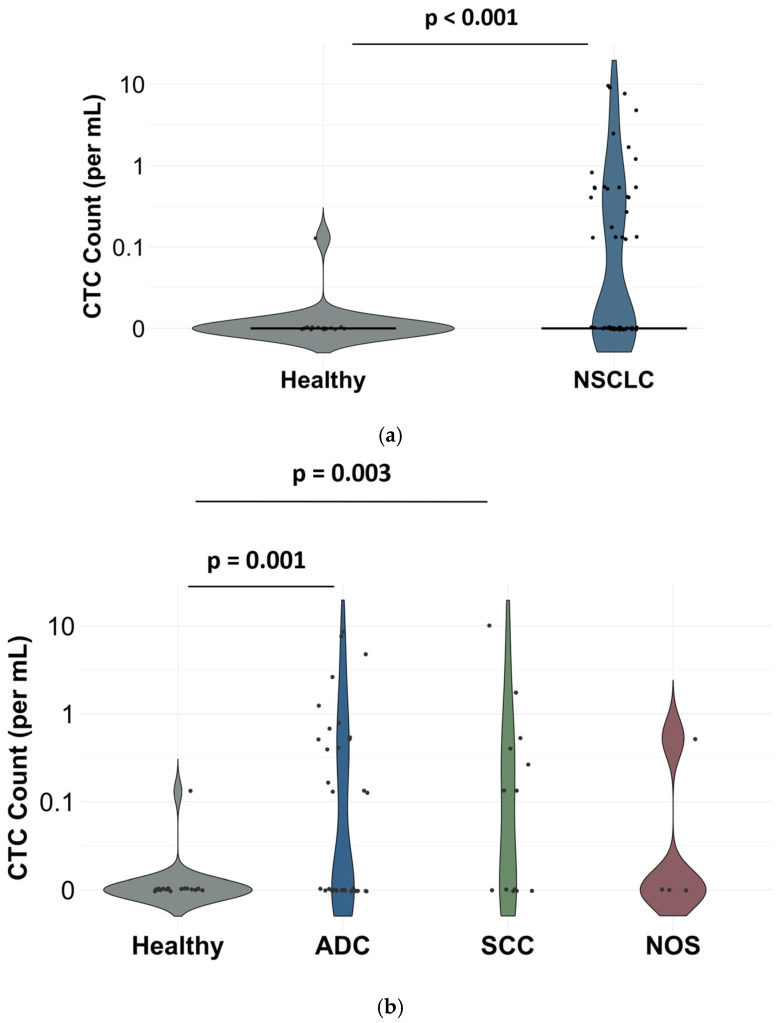
Violin plot showing the distribution of circulating tumor cell (CTC) counts per patient (**a**) in NSCLC (non-small-cell lung cancer) patients and healthy individuals. Statistical comparisons were performed using the Mann-Whitney U Test (*p* < 0.05, 95% CI) and (**b**) across NSCLC histological subtypes and healthy donors. Statistical comparisons were performed using the Wilcoxon rank-sum test (*p* < 0.05, 95% CI). Each dot represents an individual sample.

CTCs were identified by immunofluorescence staining, defined as PanCK+/EpCAM+/Vim+, CD45-negative, and DAPI-positive cells. Following SwayChip enrichment of peripheral blood samples on the SwayBox platform, enumeration was performed per milliliter of blood to correct for different blood sample input volumes. CTCs were detected in 24 of 51 NSCLC patients (47%), with counts ranging from 1 to 72 cells per patient/7.5 mL whereas only one of 20 healthy donors (5%) had a detectable CTC (Figure 3a). Among histological subtypes, positivity rates were 49% in ADC, 50% in SCC, and 25% in NOS (Table 1).

Statistical comparison demonstrated a significantly higher CTC burden in NSCLC patients compared with healthy donors (Mann–Whitney U test, *p* < 0.001). The sensitivity and specificity of the assay were 47.1% and 95.0%, respectively, when the cutoff was set at 1 CTC/7.5 mL. Subtype-specific analysis further confirmed significant differences between healthy individuals and both ADC (Wilcoxon rank test, *p* = 0.001) and SCC (*p* = 0.003) patients (Figure 3b), while no significant difference was observed between NSCLC subtypes themselves.

Regarding clinicopathological correlations, no significant associations were observed between CTC positivity and patient age (*p* = 0.427), sex (*p* = 0.759), or tumor volume (*p* = 0.594). However, CTC-positive patients exhibited a significantly higher number of metastatic organs compared with CTC-negative patients (median = 2 vs. 2; Mann–Whitney U test, *p* = 0.044), whereas the number of metastatic lymph nodes was significantly lower in CTC-positive compared with CTC-negative patients (median = 3 vs. 6; Mann–Whitney U test, *p* = 0.013) (Table 1).

**Figure 4:**
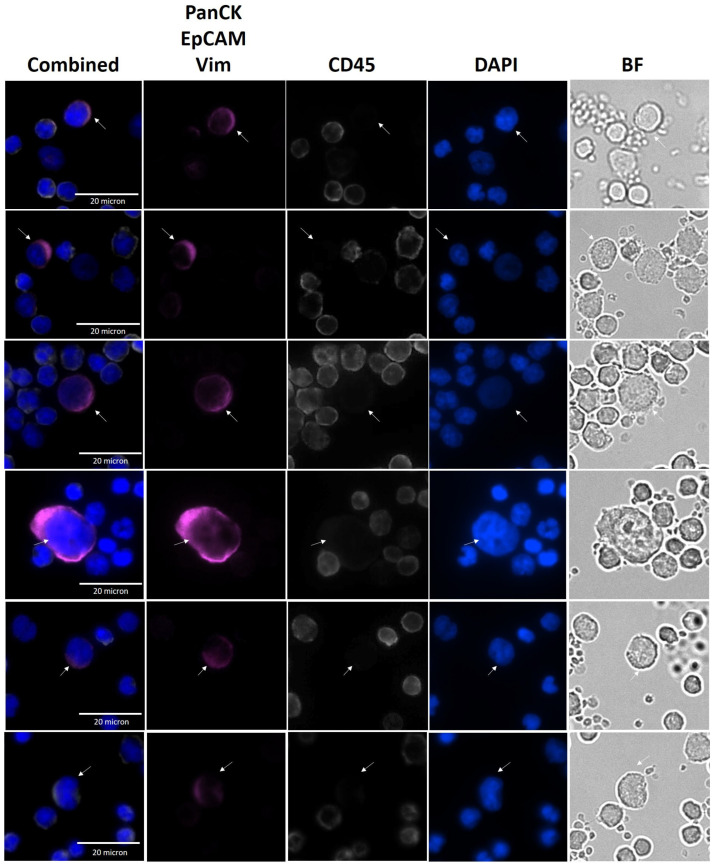
Representative images of CTCs from NSCLC patients. Immunofluorescence images showing circulating tumor cells (CTCs) enriched from patient samples. Combined and single-channel views are presented, including PanCK/EpCAM/Vim, magenta; DAPI, blue; CD45, white; and Bright Field (BF) images. White arrows indicate CTCs.

Representative images from CTCs identified in NSCLC patient samples by the SwayLights CTC identification assay are shown in Figure 4, highlighting the morphological features and staining profiles used for classification. Identification was performed by a single trained investigator to ensure consistency, and uncertain cases were independently reviewed by a second observer. The antibody cocktail and the applied IF staining protocol have been extensively optimized and validated through analytical and clinical studies to eliminate false positives and false negatives. The original representative images of CTC and WBC in Figure 4 shows clear distinction of CTCs from WBCs without background signals or nonspecific staining. In addition, the cells did not lose membrane integrity and circular morphology (WBCs) indicating cell minimal damage during hydrodynamic enrichment process.

### 3.3. Average Diameter of CTCs from NSCLC Samples in Comparison with Cell Lines

**Figure 5:**
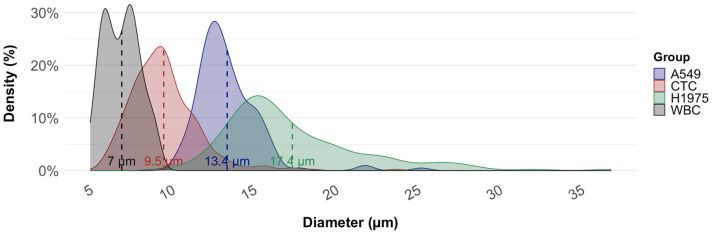
Size distribution of circulating tumor cells (CTCs), white blood cells (WBCs), and NSCLC cell lines (A549, H1975). Kernel density plots illustrate the diameter distribution of each group. Dashed vertical lines represent mean diameters: WBCs (7.0 µm), patient-derived CTCs (9.5 µm), A549 cells (13.4 µm), and H1975 cells (17.4 µm).

Since the Cellsway platform relies on size-based CTC isolation, we analyzed the diameters of CTCs obtained from patient samples and compared them with the corresponding NSCLC cultured cell lines used for analytical validation. Cell size measurements were performed following completion of the full CTC isolation and IF staining workflow, using fluorescence microscopy in combination with the Bioview image analysis software. Table 2 summarizes the mean diameters of A549 (n = 158) and H1975 (n = 152) cell lines, patient-derived WBCs (n = 100), and patient-derived CTCs (n = 321). The observed difference in mean cell diameters—17.4 ± 4.3 µm for H1975 and 13.4 ± 2.0 µm for A549—was consistent with the respective variation in recovery rates obtained for these cell lines. The mean diameter of CTCs was significantly smaller than that of both cell lines (*p* < 0.001), suggesting that patient-derived CTCs exhibit greater morphological heterogeneity than cultured cell lines. Although CTCs isolated from NSCLC patient samples exhibited smaller diameters compared to cultured cell lines, the SwayBox was able to effectively enrich CTCs by optimizing flow rates and operating parameters. In our previously published analytical study, recovery of A549 cells was 62.3% when the microfluidic chip was operated at a flow rate of 1.5 mL/min. Reducing the flow rate to 1.2 mL/min improved recovery to 78.3%, albeit with a concomitant reduction in WBC depletion efficiency (from 95.2% to 54.2%), thereby lowering the overall purity of the enriched CTC suspension. Nevertheless, for immunofluorescence (IF)-based CTC identification, this reduction in purity did not pose a significant limitation, as the absolute number of cells in the suspension remained compatible with reliable analysis on one or two microscopy slides. NSCLC CTCs size distribution partly overlapped with that of WBCs, although mean diameters differed (9.5 µm vs. 7.0 µm, respectively; Figure 5). While the distributions partly overlapped, CTCs demonstrated a modest but consistent shift toward larger diameters, enabling efficient enrichment of CTCs from WBCs. Independent WBC measurements obtained using size-calibrated fluorescent beads showed a mean diameter of 6.7 ± 0.92 µm, (N = 150) closely matching the BioView-derived WBC size estimates and confirming the reliability of our measurement approach.

## 4. Discussion

This study reports the analytical and clinical validation of the Cellsway microfluidic platform for CTC enrichment in NSCLC, using both spiked blood samples and patient blood samples alongside healthy controls. The platform isolates CTCs directly from whole blood by exploiting differences in cell size and deformability relative to blood cells.

In standardized spike-in experiments to assess analytical performance, SwayBox microfluidics achieved recovery rates of 91.9 ± 7.9% for H1975 and 78.3 ± 18.8% for A549. These recovery rates surpass those reported for both epitope-dependent and label-free technologies, using the same cell lines [54]. The higher recovery of H1975 cells can be attributed to their larger mean diameter (17.3 µm) compared to A549 cells (14.8 µm), consistent with the size-dependent separation principle of SwayBox microfluidics. This finding aligns with previous studies on size-based isolation methods, which similarly reported variable recovery efficiencies across NSCLC cell lines depending on mean cell diameter [44,46,55,56,57,58] (Table S2). In our previously published analytical study, recovery of A549 cells was 62.3% when the microfluidic chip was operated at a flow rate of 1.5 mL/min [53]. Reducing the flow rate from 1.5 mL/min to 1.2 mL/min in the current study significantly improved the recovery rate of A549, highlighting the capability of the SwayBox microfluidics in fine-tuning hydrodynamic conditions according to the clinical application and the downstream analysis to be performed. As emphasized in prior studies, preserving both morphological and molecular integrity is particularly relevant, since it enables comprehensive analyses ranging from mutational profiling to ex vivo drug sensitivity assays [59].

To evaluate the clinical performance of Cellsway platform, blood samples from 51 metastatic NSCLC patients and 20 healthy control samples have been collected and analyzed through the standardized workflow. At least one CTCs have been identified in 47% (24/51) of NSCLC patients, while only in 5% of healthy control samples (1/20). Using a threshold of 1 CTC per 7.5 mL, the assay achieved 95% specificity and 47.1% sensitivity (AUC: 0.720, *p* < 0.0001) (Figure S4). Among NSCLC subtypes, ADC and SCC were both significantly different from healthy donors (Wilcoxon *p* = 0.001 and *p* = 0.003), while no significant difference was observed between ADC and SCC, suggesting similar detectability across major NSCLC phenotypes.

Importantly, CTC positivity was significantly associated with the number of metastatic organs (*p* = 0.044), indicating that CTC detection increases with the extent of systemic spread. Although CTCs are the cellular precursors of metastases, only a very small fraction can survive circulation and colonize distant sites [60]. This positive correlation suggests that patients with multi-organ involvement have higher likelihood of CTC release into the circulation, resulting in greater shedding of tumor cells into the bloodstream. Therefore, the coexistence of multiple organ metastases and CTC positivity may represent an aggressive phenotype capable of sustaining CTC survival and spread [61]. These results highlight the potential of CTC detection not only as a diagnostic or prognostic biomarker but also as a dynamic indicator of tumor spread.

Interestingly, we observed an inverse relationship between CTC positivity and the number of metastatic lymph nodes (*p* = 0.013). This pattern may reflect distinct routes of metastatic dissemination, either through lymphatic vessels toward regional lymph nodes or directly into the bloodstream leading to distant organ colonization. While lymphatic spread represents a stepwise, localized process, hematogenous spread reflects a more aggressive, systemic progression [44,55]. In our study, CTC positivity correlated with a higher number of metastatic organs but fewer metastatic lymph nodes, may suggest a shift toward hematogenous dissemination in CTC-positive patients.

This finding, though requiring confirmation in larger patient populations, suggests that CTC burden may provide information complementary to conventional nodal staging, potentially reflecting alternative metastatic routes. This is in line with recent evidence that absolute CTC burden holds prognostic value independent of conventional staging, with baseline counts above three CTCs predicting significantly reduced overall survival [46].

Direct performance comparisons between different CTC isolation platforms remain challenging in the absence of controlled, bench-to-bench evaluations. Differences in patient cohorts, enrichment strategies, and—most importantly—the lack of standardization in sample collection, processing, and CTC identification contribute to substantial variability across studies. Furthermore, the inherent heterogeneity of tumor cells and molecular diversity among patients with the same disease add another layer of complexity, limiting the reliability of literature-based comparisons. Despite these limitations, the 47% CTC positivity rate achieved with the Cellsway platform in our NSCLC cohort aligns well with reported detection ranges of 20–60% in metastatic NSCLC across various isolation methods [46,56,57,58]. SwayBox platform demonstrated superior sensitivity compared to the EpCAM-dependent CellSearch system, with a 47% positivity rate in our 51-patient cohort versus ~30% reported for CellSearch in similar patient populations [62]. Label-free enrichment platforms have also reported variable results: ISET achieved 33% positivity, while Parsortix showed positivity rates ranging from 35% to 61% depending on the study [63,64] (Table 3). Notably, EMT-induced downregulation of cytokeratins can further impact positivity rates even in label-free systems [65,66].

The Cellsway platform combines high-recovery SwayBox microfluidics with an extensively optimized SwayLights IF staining workflow to ensure accurate CTC identification. Because variations in antibody clones, marker panels, and staining protocols are known to cause major inconsistencies across CTC platforms, rigorous standardization was essential; through systematic testing, the optimal antibody cocktail and staining conditions were defined, minimizing variability and enabling robust, reliable detection of all available CTCs with clear separation from leukocytes. Representative images of patient-derived CTCs (PanCK^+^/Vim^+^/EpCAM^+^, CD45^−^) through SwayLights clearly show the distinction between CTCs and leukocytes, with minimal background signals, nonspecific staining, and loss of cell integrity during hydrodynamic enrichment.

CTCs isolated directly from patient blood samples exhibited significantly smaller mean diameter compared to established NSCLC cell lines, and significantly larger mean diameter compared to WBCs. After enrichment and IF analysis, measured sizes of patient-derived CTCs ranged between 5.8 µm to 23.7 µm, averaging at 9.5 ± 2.0 µm. WBCs sizes averaged at 7.0 ± 1.1 µm (range: 5.1–9.2 µm); and cultured NSCLC lines were measured as 13.4 ± 2.0 µm (range: 10.3–25.2 µm) for A549; 17.4 ± 4.3 µm (range: 9.9–36.8 µm) for H1975. The observed size discrepancies between CTCs and cultured cell lines are in line with those reported by Mendelaar et al., who reanalyzed more than 71,000 CTCs across four tumor types and showed that patient-derived CTCs were consistently smaller than cultured tumor cell lines across tumor types, supporting the notion that CTCs represent a biologically distinct and heterogeneous population [73]. These finding highlights a fundamental biological distinction between patient-derived CTCs and immortalized tumor cells grown under nutrient-rich, uniform in vitro conditions. Whereas cultured cells typically display enlarged and homogeneous morphologies, CTCs circulate in vivo under shear stress, immune pressure, and metabolic constraints, which favor the emergence of smaller and more deformable phenotypes [74]. Furthermore, the close agreement between WBC diameters obtained via BioView analysis (6.7 ± 0.92 µm) and those measured independently using size-calibrated fluorescent beads provides additional methodological validation for our measurements. Notably, bead-based assessments were performed immediately after Secoll-based WBC isolation, whereas BioView-derived values were obtained following full microfluidic chip processing, fixation, and IF staining. The consistency between these two conditions indicates that neither microfluidic enrichment nor downstream processing induces measurable shifts in cell size, thereby supporting the accuracy of our CTC size measurements and confirming that the observed size differences reflect true biological variation rather than technical artifacts.

Published studies report a wide range of CTC sizes across different cancers. In NSCLC, some studies have documented mean diameters as large as 30 µm [56], whereas others observed much smaller CTCs ranging from 5–16 µm, with the majority (71%) classified as small-sized [75]. A recent report further defined small CTCs in lung cancer using a 5 µm threshold, categorizing cells below this cut-off as “small CTCs” [76]. Such variability in size is also associated with EMT–associated plasticity, where tumor cells adopt more flexible and invasive properties to enhance survival in the bloodstream and facilitate metastatic dissemination. Supporting this, Wenta et al. (2024) demonstrated significant size differences according to phenotypic state, with median diameters of ~18.2 µm for epithelial, ~12.1 µm for hybrid epithelial/mesenchymal, and ~10.4 µm for mesenchymal CTCs [74]. Our data on size distribution of CTCs for each patient, stratified by histological subtype, (presented in Figure S3), show that while average CTC size may differ across subtypes, both small and large CTCs can be recovered from the same patient sample. This observation highlights the ability of the Cellsway platform to capture CTCs across a wide size spectrum within a single blood draw, underlining its suitability for detecting the morphological heterogeneity typical of NSCLC-derived CTCs. Therefore, the reduced size of patient-derived CTCs should be interpreted not as a methodological limitation but as a biologically meaningful reflection of their heterogeneity and adaptive strategies in vivo.

In our measurements, the mean WBC size (7.0 µm) was smaller than values often reported in the literature. This difference may reflect methodological difference in image-based measurements and sample preparation (centrifugation, fixation), as well as biological composition, since PBMC fractions obtained in density gradient centrifugation are typically depleted in granulocytes, relatively larger WBCs, and enriched in lymphocytes, relatively smaller WBCs. As CTC and WBC sizes overlap due to the small-sized CTCs, size-based methods can be tuned through flow and pressure adjustments to balance recovery and purity. This was supported by our analytical results: reducing the flow rate from 1.5 mL/min to 1.2 mL/min resulted in an increase in A549 recovery. Although WBC depletion rate is compromised, the absolute cell yield remained sufficient for reliable IF review on one or two slides. Together, these findings demonstrate that although NSCLC CTCs are small and heterogeneous, sensitivity can be preserved by hydrodynamic tuning without sacrificing interpretability.

Beyond performance, workflow efficiency is crucial for adoption. In the Cellsway platform, total CTC enrichment process, including the blood pre-processing, lasts 1.5 h, of which only 12 min was the active SwayBox microfluidic enrichment step. Reported end-to-end times for other size-based hydrodynamic separation or filtration separation devices range from 1–5 h depending on the platform, for similar blood volumes (VTX-1) (VTX-1) [77,78]. Epitope-dependent isolation technologies usually require much longer incubation periods, and hands-on time, while filtration-based technologies require additional manual pre- and post-processing, reducing the efficiency, performance and usability [38].

On the other hand, the presented platform has some limitations. Although, the advantages of epitope independent enrichment are evident, small sized CTCs may not be isolated due to the size-based enrichment principle. Moreover, the WBC background may limit the applicability of the enrichment technology for single cell analysis or NGS-based downstream workflows. However, these analyses could still be performed by incorporating single cell manipulation instruments to the workflow. Finally, the SwayChip is not designed for operating with whole blood or diluted whole blood. This brings an additional pre-processing step to the workflow to remove RBCs from the blood sample, which increases the total hands-on time of the whole protocol. This could be tackled by modifying the pre-processing procedure and integrating an upstream filtering region to the microfluidic path of the SwayChip. This would significantly reduce the hands-on time and use of laboratory consumables.

## 5. Conclusions

In summary, our findings demonstrate that SwayBox microfluidic CTC enrichment technology, which employs size/deformability-based enrichment strategy, remains effective for CTC capture, even when CTCs are small and partially overlap in size with leukocytes, provided that operating conditions are carefully optimized. Being independent of surface markers, it not only enables a higher CTC yield but also a more heterogeneous CTC population compared to epitope-dependent technologies. Clinically, the platform showed a high specificity with comparable sensitivity and consistent performance across both ADC and SCC subtypes, supporting its suitability for CTC monitoring in NSCLC patients. Further studies are warranted to validate its clinical performance and demonstrate utility in real-world applications, including therapeutic response monitoring, relapse risk assessment, and biomarker identification for targeted and immunotherapies—ultimately contributing to improved survival outcomes in NSCLC and other solid tumor cancers.

## 6. Patents

The authors would like to declare the following patent associated with this research: The microfluidic CTC enrichment chip used in this study is related to the patent (US 12,036,553 B2) and is under development as a commercial product by Mikro Biyosistemler A.Ş.

## Figures and Tables

**Table 1:** Baseline demographic and clinical characteristics of NSCLC patients.

Features		N	%	N (CTC+)	CTC Positivity Rate (%)	*p*-Value
Cohort (range = 0–72)		51	100%	24	47%	
Sex						0.759
	Female	36	71%	16	44%	
	Male	15	29%	8	53%	
Age (median = 64, range = 45–82)						0.427
	≤60	15	29%	9	60%	
	>60	36	71%	15	42%	
Subtype						0.808
	ADC	35	69%	17	49%	
	SCC	12	24%	6	50%	
	NOS	4	8%	1	25%	
Lymph node metastasis						0.697
	yes	43	84%	19	44%	
	no	1	2%	1	100%	
	n/a	7	14%	4	57%	
Number of metastatic lymph node (median = 6)						**0.013**
	≤6	23	45%	14	61%	
	>6	15	29%	4	27%	
	n/a	13	25%	6	46%	
Number of metastatic organs (median = 2, range 1–4)						**0.044**
	1	3	6%	0	0%	
	2	29	57%	12	41%	
	3	10	20%	5	50%	
	4	3	6%	3	100%	
	n/a	6	12%	4	67%	
Tumor diameter (mm) (median = 32.8, range = 14–120 mm)						0.594
	≤32.8	20	39%	9	45%	
	>32.8	20	39%	8	40%	
	n/a	11	22%	7	64%	
Blood Volume						
	6 mL	6	12%	3	75%	
	6.5 mL	3	6%	18	43%	
	7.5 mL	42	82%	3	60%	
Relapse						
	yes	4	8%	3	75%	
	no	42	82%	18	43%	
	n/a	5	10%	3	60%	
Site of metastasis						
	Liver	10	20%	1	1 0%	
	Lung	8	16%	3	38%	
	Brain	8	16%	3	38%	
	Bone	25	49%	12	48%	
	Lymph node	44	86%	20	45%	
	Surrenal glands	7	14%	2	29%	
	Pleura	7	14%	3	43%	
	Neck	1	2%	0	0%	
	Cervix	1	2%	1	100%	
	Colon	1	2%	1	100%	
	Mediasten	1	2%	0	0%	
	LAP	8	16%	1	13%	

**Table 2:** Comparison of mean cell diameters of H1975, A549, WBCs and CTCs.

	Number of Cells Measure (n)	Cell Size (µm)	Std Dev	*p*-Value
H1975	152	17.4	4.3	<0.001
A549	158	13.4	2.0	<0.001
WBC	100	7.0	1.1	<0.001
NSCLC-CTC	285	9.5	2.0	-

**Table 3:** Clinical studies on NSCLC conducted using various methodological approaches.

CTC Isolation Technology	Approach	Blood Volume (mL)	Number of Patients	Number of CTC+ Patients (%)	Median (Range) CTC/mL	Reference
Microcavity assay (MCA)	Filtration based	7.5	22	17 (77)	13 (0–291)	[67]
CellSearch	EpCAM-based			7 (32)	0 (0–37)	
CellSearch	EpCAM-based	7.5	33	12 (36.4)	NA (0–19)	[68]
CellSearch	EpCAM-based	7.5	104	33 (32)	0 (0–141)	[69]
Parsortix	Size and deformability based	8	46	16 (35)	NA (1–33)	[70]
Microfluidic chip	Size-based	9	30	23 (76.6)	5 (0–16)	[71]
CellSearch	EpCAM-based	7	210	82 (39)	NA (1–23)	[72]
ISET	Size-based			104 (50)	NA (1–150)	
CellSearch	EpCAM-based	7.5	60 (stage IV)	19 (32)	1 (0–146)	[73]
Parsortix	Size and deformability based	10	15	9 (60)	NA (0–12)	[74]
ISET	Size-based			5 (33)	NA (0–3)	
Ficoll	Density gradient centrifugation			2 (13)	NA (0–2)	

## Data Availability

The original contributions presented in this study are included in the article/Supplementary Materials. Further inquiries can be directed to the corresponding author.

## Supplementary Materials

The following supporting information can be downloaded at: https://www.mdpi.com/article/10.3390/bios16010034/s1, Figure S1. Overview of the SwayBox microfluidic platform; Figure S2. Overview of the SwayBox instrument; Figure S3. CTC size distribution across patients; Figure S4. ROC Curve Analysis for NSCLC Patients and Healthy Controls; Table S1. NSCLC Patient Cohort Overview; Table S2. Analytical data reported by different methodological approaches using different cell lines.
