# Supplementary material for: Evaluation of the Cellsway Microfluidic CTC Enrichment and Identification Platform for CTC Detection in Metastatic NSCLC
Source: Biosensors (Basel). 2026 Jan 2;16(1):34. doi: 10.3390/bios16010034 (PMC12839279; doi:10.3390/bios16010034)
Supplement: Supplementary file 1 [file biosensors-16-00034-s001.zip › biosensors-3976418-supplementary.pdf]

Figure S1. Overview of the SwayChip

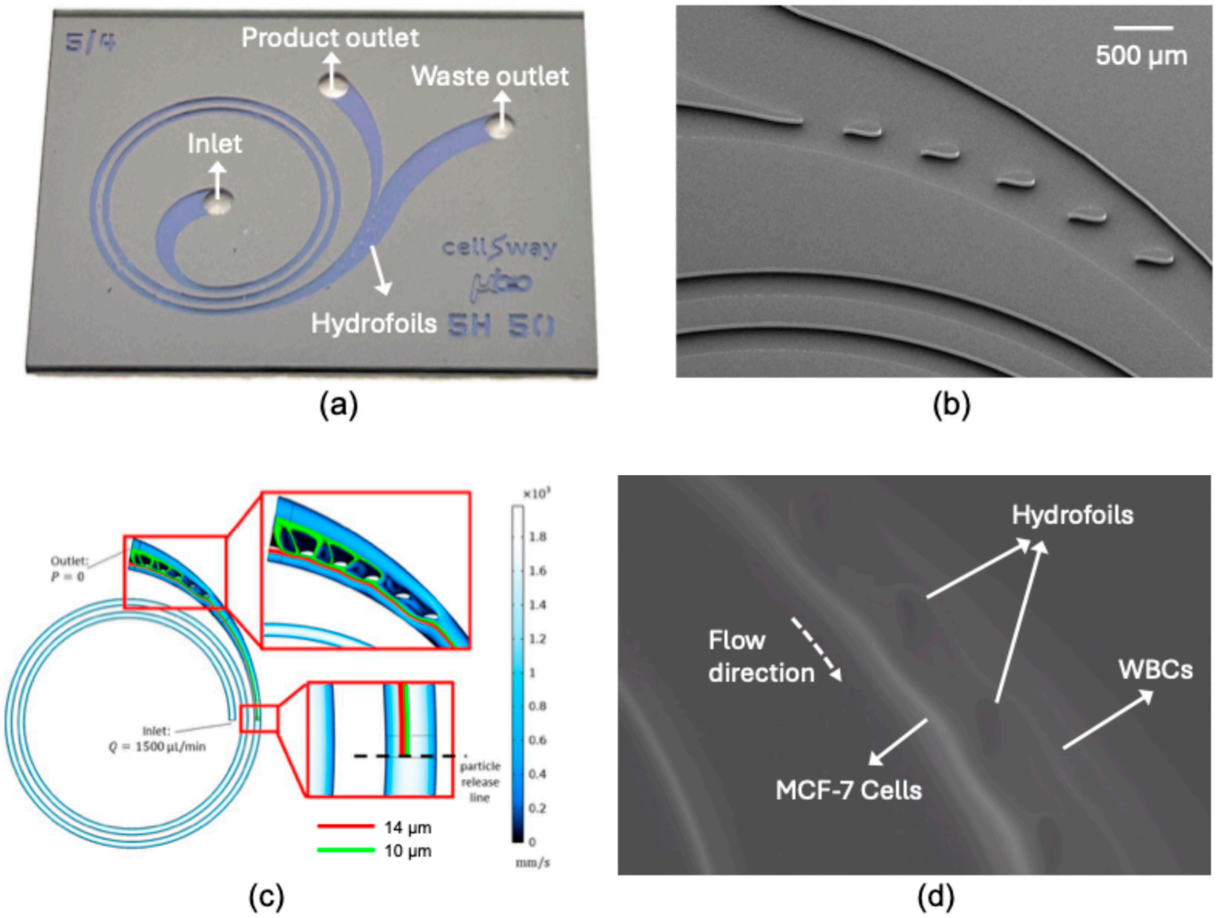

Figure S1. (**a**) The photograph of the fabricated microfluidic chip. A silicon wafer houses the microfluidic channel and the inlet and outlet ports, and the Si wafer is bonded to a blank glass wafer. The chip size is 1.65 cm x 2.15 cm. (**b**) An SEM image of the hydrofoil region and the wall separating the Product and Waste outlets. (**c**) Simulation results showing the separation of 10 µm diameter particles from 14 µm particles [49]. (**d**) Different focusing lines of fluorescently stained MCF-7 cells and WBCs inside the SwayChip. The image is generated by overlaying two images of the same region showing the focusing lines of MCF-7 cells and WBCs separately.

Figure S2. Overview of the SwayBox instrument

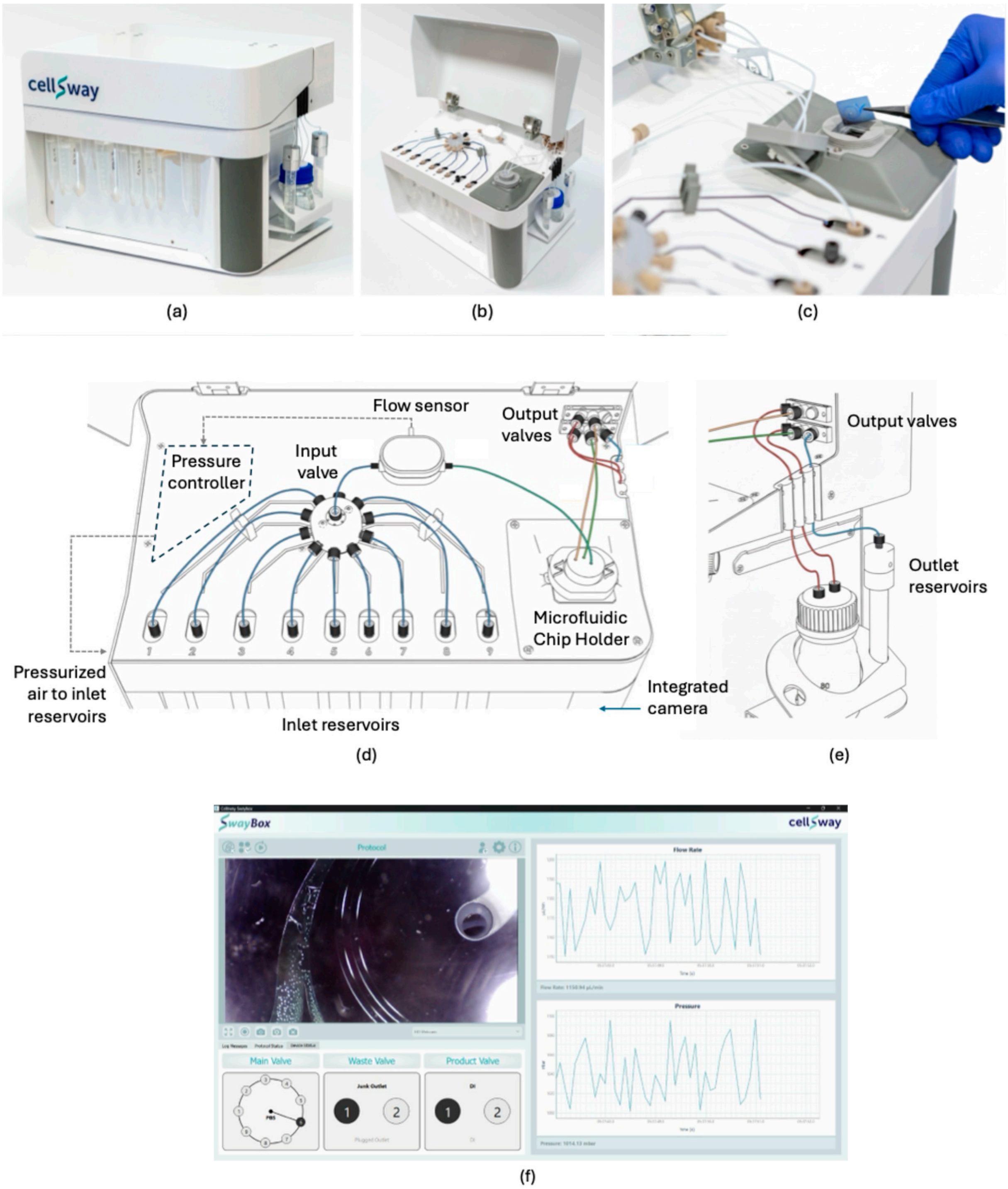

Figure S2. Overview of the SwayBox instrument. (**a**) External view, (**b**) Open-lid view showing the valves, tubing and external components. (**c**) Loading of the microfluidic chip onto the device. (**d**–**e**) Schematic representation of SwayBox components and control mechanism. SwayBox is a microfluidic platform equipped with multi-port input valve for sequential fluid delivery to SwayChip driven by a pressure source which is regulated via a flow sensor. Microfluidic chip is loaded onto SwayBox with a screw-cap holder. An integrated camera enables real-time monitoring of flow during operation. Outlet valves direct fluids into desired outlet reservoirs for collection. (**f**) The instrument is run by a custom software that automates the whole CTC enrichment protocol including chip pre-conditioning, sample processing, and post-process cleaning.

Table S1: NSCLC Patient Cohort Overview

Table S1. NSCLC Patient Cohort Overview.

Patient No	Age	Gender	Tumor Diameter (mm)	# of Metastatic Organs	# of Metastatic Lymph Node	CTC Count
**1**	63	M	58.5	3	5	0
**2**	60	F	43.5	2	0	9
**3**	64	M		2	3	6
**4**	74	M	14	2	5	1
**5**	73	M	120	2	16	0
**6**	64	M	29.5	2	12	1
**7**	67	M	53	2	6	0
**8**	70	F	24.5	2	6	0
**9**	55	M	16	2	8	1
**10**	60	M	30.5	3	6	1
**11**	60	F	13	1	9	0
**12**	78	M	40.5	2	3	4
**13**	47	M	58.5	3	6	4
**14**	69	F	77.5	2	3	4
**15**	52	F	21	2	3	37
**16**	59	F	17	3	9	0
**17**	74	M	15.5	3	8	0
**18**	64	M	20	2	5	0
**19**	60	M	55	2	7	0
**20**	62	M	44	2	7	0
**21**	81	M	18	2	9	0
**22**	51	F	35	2	8	0
**23**	61	F				2
**24**	64	M	38.5	2	6	0
**25**	72	F	20	2	5	0
**26**	56	M	61	2	8	3
**27**	52	F	25	2	6	56
**28**	69	M	30	2	6	0
**29**	51	M	42.5	2	5	0
**30**	63	M		3	1	0
**31**	62	M			3	74
**32**	67	M	55	2	3	1
**33**	48	M	19	4	3	3
**34**	82	F	45	4	1	67
**35**	67	M	26	4	2	4
**36**	72	M	15	2		0
**37**	76	M	30	2		3
**38**	72	M	29	2		0
**39**	64	M	56	2		0
**40**	70	F	35	1	multiple	0
**41**	67	M	46.5	3		0
**42**	45	F	46.5	2	multiple	0
**43**	66	M	25	1	multiple	0
**44**	75	M	56	3	multiple	1
**45**	69	M				0
**46**	68	F				4
**47**	68	M				0
**48**	58	M				4
**49**	61	F		3		19
**50**	75	M		3		13
**51**	75	M		2		0

Figure S3. CTC size distribution across patients

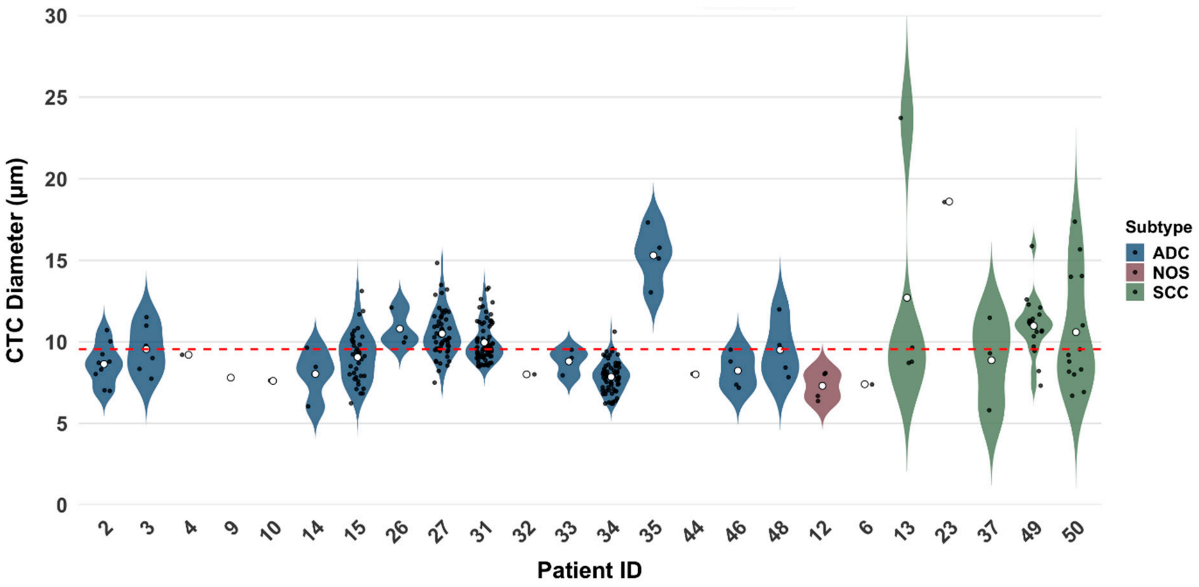

Figure S3. CTC size distribution across patients. Violin plots display the distribution of circulating tumor cell (CTC) diameters per patient, colored by histological subtype (ADC, NOS, SCC). Each dot represents the diameter of an individual CTC. The red dashed line indicates the overall mean CTC diameter (9.4 µm).

Figure S4. ROC Curve Analysis for NSCLC Patients and Healthy Controls

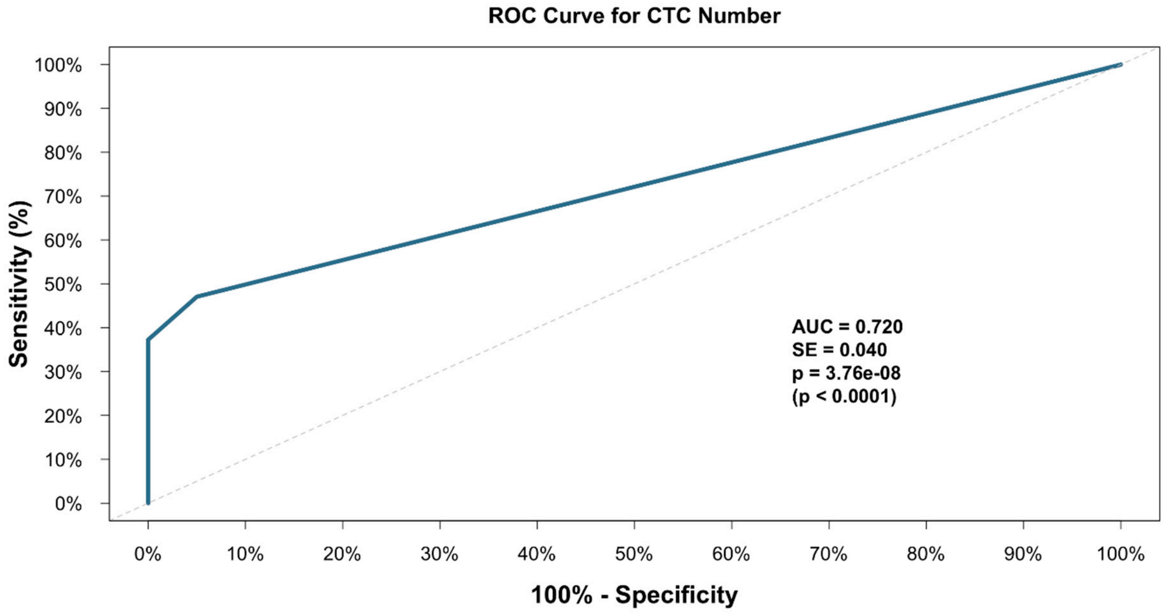

Figure S4. ROC analysis for distinguishing NSCLC patients from healthy controls based on CTC counts (per mL). The ROC curve illustrates the discriminative ability of CTC counts, with the diagonal line representing the no-discrimination (random guess) reference and the blue line indicating the ROC curve (AUC: 0.720, $p<0.0001$).

Table S2. Analytica data reported by different methodological approaches using different cell lines

Table S2. Analytical data reported by different methodological approaches using different cell lines.

CTC Isolation Technology	Approach	Cell Line	Cell Line Diameter (μm)	Recovery Rate (%)	Reference
Parsortix PR1	Size and deformability based	H1975<br>A549<br>H1299	NA	H1975: 49 ± 2<br>A549: 47 ± 10<br>H1299: 52 ± 10	[1]
EasySep	Blood cell depletion			H1975: 18 ± 8	
Rosette-Sep				H1975:25 ± 10	
The CellMag	EpCAM-dependent			H1975: 70 ± 14<br>A549: 35 ± 14<br>H1299: 1 ± 1	
Microcavity array (MCA)	Filtration based	A549<br>HCC827<br>NCI-H358<br>NCI-H441<br>PC-14	17.3<br>19.6<br>18.1<br>20.6<br>19.5	98 ± 3<br>99 ± 6<br>100 ± 6<br>98 ± 8<br>97 ± 2	[2]
Multi-flow microfluidic device	Size based	H460	NA	> 93 for spiked cell numbers > 50	[3]
Parsortix	Size and deformability based	A549<br>H1975<br>SKMES-1	NA	A549: 87 ± 5<br>H1975: 57 ± 11<br>SKMES-1: 57 ± 16	[4]
ISET	Size-based			A549: 44 ± 10<br>H1975: 55 ± 12<br>SKMES-1: 59 ± 10	

References

1. M Saini, V.; Oner, E.; Ward, M.P.; Hurley, S.; Henderson, B.D.; Lewis, F.; Finn, S.P.; Fitzmaurice, G.J.; O'Leary, J.J.; O'Toole, S.; et al. A Comparative Study of Circulating Tumor Cell Isolation and Enumeration Technologies in Lung Cancer. *Mol Oncol* **2025**, *19*, 2014–2037, doi:https://doi.org/10.1002/1878-0261.13705.
2. Hosokawa, M.; Kenmotsu, H.; Koh, Y.; Yoshino, T.; Yoshikawa, T.; Naito, T.; Takahashi, T.; Murakami, H.; Nakamura, Y.; Tsuya, A.; et al. Size-Based Isolation of Circulating Tumor Cells in Lung Cancer Patients Using a Microcavity Array System. *PLoS One* **2013**, *8*, doi:10.1371/journal.pone.0067466.
3. Zhou, J.; Kulasinghe, A.; Papautsky, I.; Bogseth, A.; Byrne, K.O.; Punyadeera, C. Isolation of Circulating Tumor Cells in Non-Small-Cell-Lung-Cancer Patients Using a Multi- Fl Ow Micro Fl Uidic Channel. *Microsyst Nanoeng* **2019**, doi:10.1038/s41378-019-0045-6.
4. Papadaki, M.A.; Sotiriou, A.I.; Vasilopoulou, C.; Filika, M.; Aggouraki, D.; Tsoulfas, P.G.; Apostolopoulou, C.A.; Rounis, K.; Mavroudis, D.; Agelaki, S. Optimization of the Enrichment of Circulating Tumor Cells for Downstream Phenotypic Analysis in Patients with Non-Small Cell Lung Cancer Treated with Anti-Pd-1 Immunotherapy. *Cancers (Basel)* **2020**, *12*, 1–26, doi:10.3390/cancers12061556.
